# Neuronal activity in dorsomedial and dorsolateral striatum under the requirement for temporal credit assignment

**DOI:** 10.1038/srep27056

**Published:** 2016-06-01

**Authors:** Eun Sil Her, Namjung Huh, Jieun Kim, Min Whan Jung

**Affiliations:** 1 Center for Synaptic Brain Dysfunctions, Institute for Basic Science, Daejeon 34141, Korea; 2 Neuroscience Graduate Program, Ajou University School of Medicine, Suwon 16499, Korea.; 3 Physician-Scientist Training Program, Ajou University School of Medicine, Suwon 16499, Korea; 4 Department of Biological Sciences, Korea Advanced Institute of Science and Technology, Daejeon 34141, Korea

## Abstract

To investigate neural processes underlying temporal credit assignment in the striatum, we recorded neuronal activity in the dorsomedial and dorsolateral striatum (DMS and DLS, respectively) of rats performing a dynamic foraging task in which a choice has to be remembered until its outcome is revealed for correct credit assignment. Choice signals appeared sequentially, initially in the DMS and then in the DLS, and they were combined with action value and reward signals in the DLS when choice outcome was revealed. Unlike in conventional dynamic foraging tasks, neural signals for chosen value were elevated in neither brain structure. These results suggest that dynamics of striatal neural signals related to evaluating choice outcome might differ drastically depending on the requirement for temporal credit assignment. In a behavioral context requiring temporal credit assignment, the DLS, but not the DMS, might be in charge of updating the value of chosen action by integrating choice, action value, and reward signals together.

Attributing an outcome to its causal action can be trivial when sensory information regarding the chosen action is available at the time that its outcome is revealed. Often, however, the consequence of an action is substantially delayed, and sensory information regarding the chosen action is unavailable when its outcome is revealed. In this case, one needs a memory trace to correctly attribute the outcome to its causal action. This is referred to as the temporal credit assignment problem^1^, which humans and animals encounter frequently in their daily lives. Despite its importance, however, its underlying neural mechanisms are poorly understood. Although a large body of studies has investigated neural processes related to action-outcome association and updating action value based on experienced outcomes^2^,^3^,^4^,^5^,^6^,^7^, sensory information regarding chosen action, including proprioceptive information in many cases, was available when its outcome was revealed in almost all studies so far. This is in contrast to a large number of studies using the trace conditioning paradigm in which a sensory cue is paired with an outcome over a temporal delay. As a result, we have limited understanding on neural mechanisms of attributing an outcome to its causal action across a temporal gap.

We investigated this issue in the dorsal striatum by employing a novel behavioral task for rats in which sensory information regarding a chosen action is unavailable when its outcome is revealed. We have shown previously that choice signals persist differently in the dorsomedial (associative) and dorsolateral (sensorimotor) striatum (DMS and DLS, respectively) in rats. Specifically, choice signals persisted until the outcome of the next goal choice was revealed in the DMS, but not the DLS, in a dynamic foraging task in which keeping track of choices and their outcomes over multiple past trials was advantageous (‘dual assignment with hold’ task; reward probability increased as a function of consecutive alternative choices)^8^. This finding is consistent with numerous behavioral studies indicating roles of the DMS and DLS in response-outcome and stimulus-response associations, respectively^9^,^10^,^11^,^12^,^13^. We therefore expected that persistent choice signals that are necessary to attribute an outcome to its causal action would be carried by the DMS, but not the DLS, in the present behavioral task. To our surprise, choice signals appeared sequentially, initially in the DMS and then in the DLS. Moreover, neural signals necessary to update the value of the chosen action converged in the DLS, but not the DMS, when the choice outcome was revealed. These results suggest that striatal neural processes underlying the evaluation of choice outcome vary widely depending on the requirement for temporal credit assignment.

## Results

### Behavior

**Figure 1:**
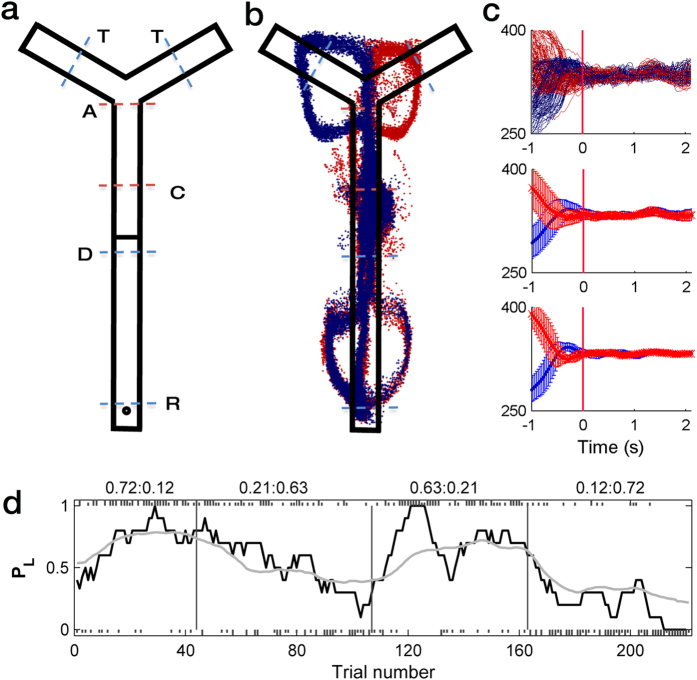
Animal behavior. (**a**) Behavioral task. In each trial, following a 2-s delay at the delay point (D), the animal was required to choose either the left or right target (T) by checking a photobeam sensor (blue dashed lines on top), return to the reward location (R, circle), and wait for 1 s to obtain the water reward. Approximate spatial positions for the divergence (outbound) and convergence (inbound) of left- and right-choice-associated movement trajectories, which were determined separately for each session, are indicated as A (approach) and C (convergence), respectively (red dashed lines). (**b**) An example of movement trajectory for one session. Blue, left choice; red, right choice. Each dot represents the animal’s head position at 33.3 ms time resolution. (**c**) Determination of the convergence point. X-coordinates of the animal’s head position data were temporally aligned to the time point 3 s prior to the reward stage onset. The first time point when the difference in X-coordinates of the left- and right-choice trials became statistically insignificant (*t*-test, *p* > 0.05) and remained that way for at least nine consecutive points (300 ms) was determined as the convergence point (red vertical line). Top, X-coordinates of all left-choice and right-choice trials in an example session; middle, mean (±SD across trials) X-coordinates of the left-choice and right-choice trials of the same session; bottom, mean X-coordinates of the left-choice and right-choice trials were averaged across sessions (±SD). The data was aligned to the convergence point (time 0) that was determined separately for each session. (**d**) Choice behavior of one animal in one example session. Tick marks denote trial-by-trial choices of the animal (upper, left choice; lower, right choice; long, rewarded trial; short, unrewarded trial). Vertical lines denote block transitions and numbers on the top indicate mean reward probabilities associated with left and right choices in each block. The black line shows the probability to choose the left target (*P*_*L*_) in a moving average of 10 trials, and the gray line shows *P*_*L*_ predicted by the hybrid model.

Three rats were trained to perform a dynamic two-armed bandit (TAB) task on a Y-shaped maze (Fig. 1a). The animals were free to choose between two targets (left vs. right). Each choice was associated with different reward probabilities (12, 21, 63 or 72%) that were constant within a block of 40–75 trials (54.2 ± 6.5, mean ± SD), but changed across four blocks. No sensory cue was available to the animals for the block change so that the animals had to discover changes in reward probabilities by trial and error. A major difference from our previous dynamic foraging tasks^6^,^14^,^15^,^16^,^17^ was that reward was delivered not at the target locations, but after the animal went to the opposite end of the maze (reward location; ‘R’ in Fig. 1a) and waited for 1 s. Thus, no sensory information about the animal’s choice was available when the choice outcome was revealed. In this task, therefore, the animals had to retain the information on the latest choice until its outcome was revealed in order to correctly associate a choice and its consequence (temporal credit assignment problem^1^). Figure 1b shows movement trajectories of one animal during one example session. In all sessions, as shown in the example, the animal’s movement trajectories following left- vs. right-choices converged (Fig. 1c) around the midpoint of the central stem (‘C’ in Fig. 1a). The duration of the memory stage (the time period between the convergence of the animal’s movement trajectories and trial outcome), during which the animals had to retain the information regarding the chosen action for correct temporal credit assignment, was 1.92 ± 0.13 s (mean ± SD).

The animals completed the majority of the trials. The number of incomplete trials (returning to the reward location without checking a target sensor) was 1.7 ± 0.3 (0.8 ± 0.1%; mean ± SEM) per session. During the steady state (i.e., the last 10 trials in each block), the animals chose the higher reward-probability target in 62.8 ± 2.0% of the trials (comparison with 50% choice, *p* = 3.2 × 10^−7^; 67.1 ± 2.5% in 0.72:0.12 blocks and 58.4 ± 2.2% in 0.63:0.21 blocks). This value is significantly lower compared to that in a conventional dynamic TAB task^15^ (75.6 ± 7.9%; *t*-test, *p* = 2.9 × 10^−8^). Thus, the animals captured changes in reward probabilities and adjusted their choices accordingly even with a delay between a choice and its outcome (Fig. 1d), although their performance (in terms of choosing the higher reward-probability target) was significantly lower than in the conventional dynamic TAB task in which choice outcome was revealed at target locations.

### Temporal profiles of neuronal activity

**Figure 2:**
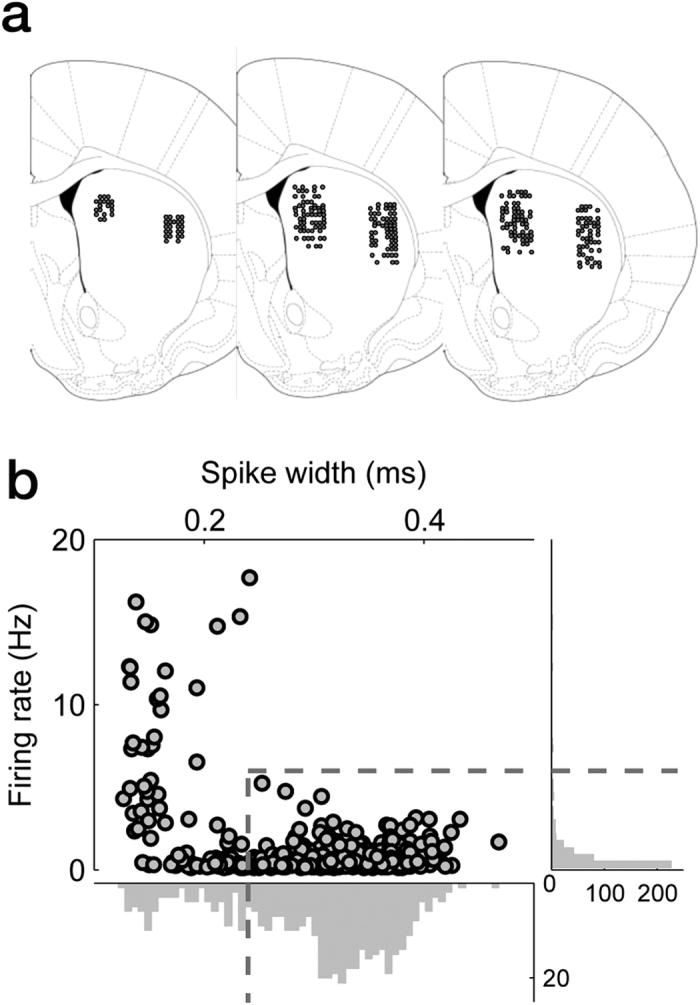
Recording locations and unit classification. (**a**) Single units were recorded from the DMS and the DLS. The diagrams are coronal section views of three rat brains at 0.48 mm anterior to bregma. Each diagram represents one rat and each circle represents one recording site that was determined based on histology and electrode advancement history. One to six units were recorded simultaneously from each site. Modified from ref. 49 with permission from Elsevier. (**b**) Unit classification. Recorded units were classified into putative MSNs and putative interneurons based on mean discharge rate and spike width. Those units with mean firing rates <6 Hz and spike widths ≥0.24 ms were classified as putative MSNs, and the rest were classified as putative interneurons.

**Figure 3:**
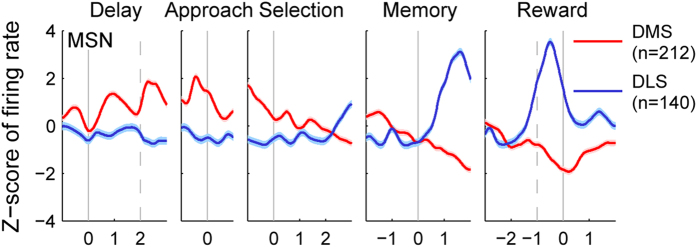
Temporal profiles of neuronal activity. Mean discharge rates of putative MSNs were compared between the DMS (red) and DLS (blue). The graphs show z-scores of discharge rates of putative MSNs across different behavioral stages (delay, approach to target, target selection, memory, and reward). A spike density function was constructed for each neuron by applying a Gaussian kernel (σ = 100 ms) and then z-normalized based on the mean and SD of discharge rate in 10-ms time bins. Shading indicates 95% confidence interval. Each solid vertical line indicates the beginning of a given behavioral stage. Dashed vertical lines denote delay stage offset (left) and the animal’s arrival at the reward location (right).

A total of 328 and 204 single units were recorded from the right DMS and DLS, respectively, from three rats (Fig. 2a). The recorded units were classified into putative medium spiny neurons (MSNs; DMS, n = 212; DLS, n = 140) and putative interneurons (DMS, n = 49; DLS, n = 41) based on mean discharge rates and spike widths^8^ (Fig. 2b), and we focused our analysis on putative MSNs. Activity of the putative MSNs (mean of *z*-normalized discharge rate) was higher initially in the DMS, but in the DLS in the later phase of a trial (Fig. 3). DMS neurons gradually decreased their activity from the approach stage onset (see Methods for behavioral stages) until the reward stage onset, whereas DLS neurons rapidly increased their activity starting from the memory stage onset, which reached a peak at ~0.5 s before the reward stage onset (Fig. 3). The elevated DLS activity might be related to reward anticipation, such as the activity found in the ventral striatum^18^. Overall, DMS neurons showed higher activity before the memory stage onset, whereas DLS neurons showed higher activity after the memory stage onset, raising a possibility that the DMS and DLS might play more important roles in the early and late phases of a trial, respectively, in the present behavioral task.

### Neural signals for choice and reward

**Figure 4:**
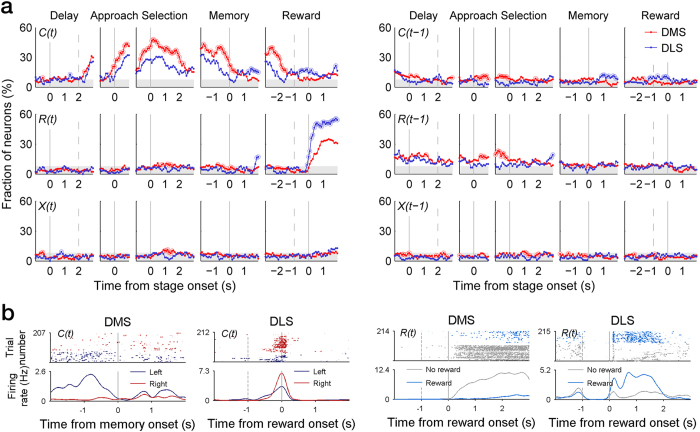
Neural activity related to the animal’s choice and its outcome. (**a**) Shown are fractions of DMS and DLS MSNs that significantly modulated their activity according to the animal’s choice (*C*), its outcome (*R*), and their interaction (*X*) in the current (*t*) and previous (*t* − 1) trials in a 500-ms time window that was advanced in 100-ms time steps across different behavioral stages. Shading indicates chance level (binomial test, alpha = 0.05) for the DLS (7.86%), which is slightly higher than that for the DMS (7.55%). Large open circles indicate significantly different fractions (*χ*^2^-test, *p* < 0.05) between DMS and DLS. Behavioral stages and vertical lines are as shown in Fig. 3. (**b**) Examples of MSNs responsive to animal’s choice (*C*) or its outcome (*R*) in the current trial (*t*). Top, spike raster plots. Each row is one trial, and each dot represents a spike. Bottom, spike density functions. Trials were divided into two groups according to the animal’s target choice (left vs. right) or reward (rewarded vs. unrewarded).

We examined neural signals related to the animal’s choice (*C*), its outcome (i.e., reward; *R*), and their interaction (*X*) in the current (*t*) and two previous trials (*t* − 1 and *t* − 2) in a 500-ms time window that was advanced in 100-ms time steps based on a multiple linear regression analysis (see Methods). Figure 4a shows fractions of neurons significantly responsive to these behavioral variables [*C*(*t* − 2)*, R*(*t* − 2)*, and X*(*t* − 2) data are not shown because they were largely below chance level]. Advanced choice signals were weak in both DMS and DLS as reported previously^8^,^14^. Choice signals were elevated following the approach onset and stayed well above chance level in both brain structures, but were stronger in the DMS than the DLS. Once the animal passed through the convergence point (memory stage onset), however, DMS choice signals gradually decayed so that they fluctuated near chance level before and after the reward stage onset. By contrast, DLS choice signals initially went down below chance level after trajectory convergence, but arose above chance level ~700 ms before the animal arrived at the reward location and stayed well above chance level until ~1 s following the reward stage onset (Fig. 4a). Thus, choice signals were initially carried by the DMS after trajectory convergence, but the DLS took over this role as the animal reached the reward location.

Reward signals arose immediately after the reward stage onset in both areas, but the magnitude (i.e., fraction of neurons) was larger in the DLS than the DMS, which is consistent with a previous finding^19^. Examples of choice- and reward-responsive neurons are shown in Fig. 4b.

Previous choice signals were much weaker, but showed a similar trend (Fig. 4a). Previous choice signals were stronger in the DMS than the DLS in the early phase of a trial (early delay and approach stages), but stronger in the DLS than the DMS in the late phase (~0.5 s following the memory stage onset). Previous reward signals were somewhat stronger than previous choice signals. They were maintained above chance level until the memory stage onset in the DMS and until the approach stage onset in the DLS. Previous reward signals were significantly stronger in the DMS than the DLS, especially during the approach stage.

### Neural signals for value

**Figure 5:**
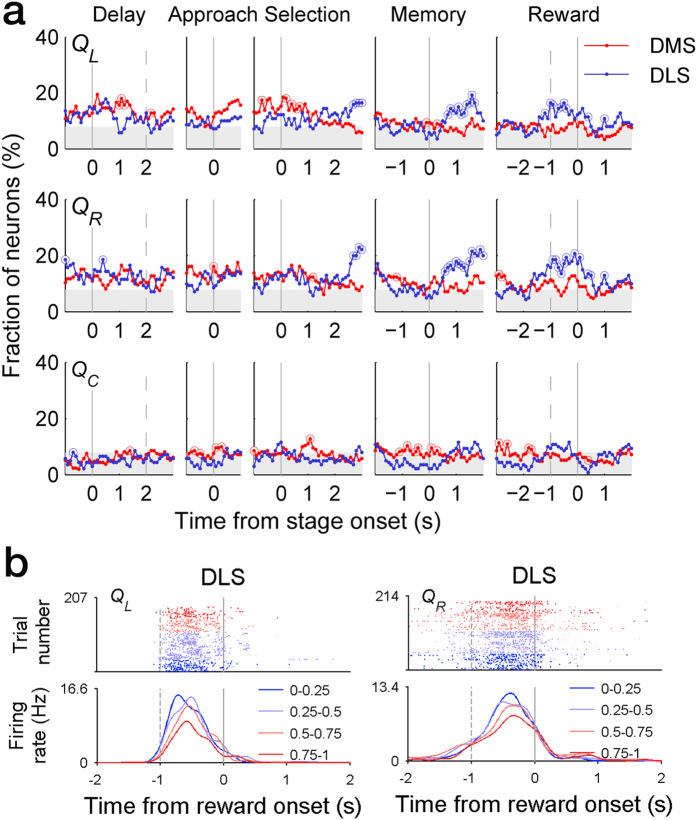
Temporal profiles of value-related neural activity. (**a**) Shown are fractions of DMS and DLS MSNs that significantly modulated their activity according to left action value (*Q*_*L*_), right action value (*Q*_*R*_), and chosen value (*Q*_*C*_). Analysis time windows and shading are as shown in Fig. 4a. (**b**) Two examples of DLS MSNs coding action value. Top, spike raster plots. Bottom, spike density functions. Trials were divided into four groups according to the level of *Q*_*L*_ (left) or *Q*_*R*_ (right).

Neural signals for action value (*Q*_*L*_ and *Q*_*R*_), that was estimated with a reinforcement learning (RL) model, were overall weak before the animal returned to the reward site. However, action value signals in the DLS, but not the DMS, increased as the animal passed the convergence point and approached the reward location (~0.5 s after passing the convergence point). The elevated action value signals subsided ~1 s following the reward stage onset (Fig. 5a). Analyzing the 2-s time window centered around the reward stage onset, the fraction of neurons conveying at least one action value signal (*Q*_*L*_ or *Q*_*R*_; *t*-test, *p* < 0.025) was significantly higher in the DLS than the DMS (20.7 and 9.0%, respectively; *χ*^2^-test, *p* = 0.002). Chosen value (*Q*_*c*_) signals were much weaker than action value signals throughout the task. Examples of value-responsive neurons are shown in Fig. 5b.

## Discussion

To investigate neural processes underlying temporal credit assignment in the striatum, we recorded DMS and DLS neuronal activity in a behavioral task in which chosen action has to be remembered until its outcome is revealed for correct attribution of the outcome to its causal action. To our surprise, DMS and DLS were sequentially engaged in transmitting neural signals necessary to update the value of the chosen action. Choice signals were initially transmitted by the DMS, and subsequently by the DLS during the memory stage. Choice signals in the DLS were then combined with action value and reward signals after the outcome of the choice was revealed.

Our results indicate that striatal information processing related to action outcome evaluation can be drastically different depending on the requirement for temporal credit assignment. Sensory information regarding chosen action was available when its outcome was revealed in our previous dynamic foraging tasks^8^,^14^, and, not surprisingly, both DMS and DLS conveyed strong choice signals following the animal’s choice until the reward period. In these studies, chosen value signals arose as the animals approached the target locations and stayed above chance level for ~1 s after the choice outcome was revealed in both DMS and DLS, so that they were combined with reward signals that arose steeply as soon as the choice outcome was revealed. These results suggest that both DMS and DLS were involved in updating the value of the chosen action in our previous dynamic foraging tasks^19^,^20^. The present results differ from our previous ones with respect to action evaluation in two major respects. First, neural signals necessary for value updating converged in the DLS, but not the DMS. There has been considerable interest in the roles of DMS and DLS in reward-based learning and decision making^21^,^22^,^23^. In particular, the DLS and DMS have been proposed to play roles in stimulus-response (habit formation) and response-outcome (goal-directed action selection) associations, respectively^9^,^10^,^11^,^12^,^13^,^24^,^25^. However, our results suggest that, under certain conditions that require temporal credit assignment, it is the DLS where signals necessary for response-outcome association converge. Note that this finding does not necessarily indicate the role of the DLS in response-outcome rather than stimulus-response association. Stimulus, response, and outcome signals are all necessary in order to strengthen (or weaken) stimulus-response association according to an outcome. The choice and outcome signals found in the DLS during the reward stage might be used for stimulus-response association.

The second major difference is that action value signals, instead of chosen value signals, were elevated at the time that choice outcome was revealed in the present study. Unlike in our previous studies^8^,^14^, strong chosen value signals were not observed when the choice outcome was revealed in the present study. The lack of strong chosen value signals might be an indication that neither the DMS nor the DLS is involved in updating the value of the chosen action in the present behavioral task. It is worth noting, however, that all the information necessary to update the value of the chosen action was available in the DLS when the choice outcome was revealed. Also, in computational models of reward-based learning in the striatum, striatal neurons most often do not directly represent the value of the chosen action but represent the value of a fixed specific action (e.g., refs 26,27). Thus, an alternative possibility is that the DLS updates the value of the chosen action by integrating neural signals for the animal’s choice, action values, and reward in the present behavioral task. Neural processes for updating action value might vary depending on the availability of sensory information regarding the chosen action at the time that the choice outcome is revealed. When sensory information concerning the chosen action is available, it might selectively activate those striatal neurons encoding the value of the chosen (but not the unchosen) action, so that the chosen value and reward signals are combined to update the value of the chosen action. When this is not the case, all action value signals might arise and be integrated with “maintained” choice signals and reward signals. Striatum might selectively update the value of the chosen (but not the unchosen) action based on the combination of choice, action value, and reward signals when sensory information on the chosen action is unavailable at the time that the choice outcome is revealed.

Neural processes enabling temporal credit assignment are not yet well understood^28^,^29^. Temporal credit assignment might be achieved by “tagging” synapses involved in action selection and modifying them later^28^,^30^. This might also be achieved by persistently maintaining the activity of those neurons involved in action selection until its outcome is revealed^6^,^31^,^32^. Previous studies have found prolonged choice signals for action-outcome association in the form of persistent neural activity in different parts of the brain, including various sub-regions of the frontal cortex, striatum, and hippocampus^6^,^31^. However, these studies employed behavioral tasks in which sensory information regarding the chosen action was available when its outcome was revealed, and hence were not designed to study the temporal credit assignment problem. For choice signals to be qualified as potential memory (or eligibility trace) signals for temporal credit assignment, persistent choice signals correlated with behavioral requirement of temporal credit assignment would be necessary. In this respect, we found sequentially appearing choice signals in the DMS and DLS during the time period the animal’s choice has to be remembered for correct credit assignment. It is possible that the sequentially appearing striatal choice signals have nothing to do with linking temporally discontiguous actions and their outcomes. For example, they might passively reflect neural activity in upstream brain structures (e.g., frontal cortices) that might be in charge of maintaining choice signals for temporal credit assignment. Nevertheless, our results raise the possibility that the DMS and DLS cooperate in transmitting choice signals during the memory stage for the purpose of temporal credit assignment. It has long been thought that the cortico-basal ganglia circuit consists of multiple parallel loops that serve distinct functions^12^,^33^,^34^. However, new anatomical studies indicate substantial cross-talk between different loops^35^,^36^,^37^,^38^ and physiological evidence for reciprocal interactions between the loops has been reported^39^,^40^. Our results raise the possibility that different cortico-striatal loops might cooperate under certain circumstances. Additional studies, such as employing specific manipulations of the DMS and the DLS in a task similar to the current one, might provide useful information on this issue.

It is notable that the DMS conveyed persistent choice signals until the reward stage in the next trial when it was advantageous for the animals to keep track of past choices and their outcomes (dual assignment with hold task^8^). We also found weak, but significantly stronger, DMS choice signals that persisted until the next trial in the current study (Fig. 4a), which is consistent with our previous findings^14^. Thus, the DMS tended to convey choice signals for a prolonged period across trials, especially when the information was necessary for optimal performance^8^. These results suggest that there might exist multiple mechanisms for temporal credit assignment. For linking a chosen action and its direct consequence over a temporal delay, as in the current behavioral setting, the DMS might cooperate with the DLS for action value updating. For linking a chosen action to its indirect consequences across multiple trials, as in the behavioral task used in our previous study (dual assignment with hold task^8^), the DMS might be in charge of maintaining choice information for a prolonged period. It has been proposed that different parts of the striatum concern reward prediction^41^ and action selection^42^ at different time scales. Clearly, further studies are needed to elucidate the roles of the DMS and the DLS (and other structures that are known to convey previous choice signals, as well) in temporal credit assignment under different behavioral settings.

Discharge rates of DMS and DLS neurons were higher in the early and late phases of a trial (before and after memory stage onset), respectively. These results raise the possibility that the DMS and DLS might play more important roles in different phases of a trial. Choice and action value signals were stronger in the DMS in the early phase of a trial and, conversely, they were stronger in the DLS in the late phase of a trial. Higher firing of DLS neurons in the late phase of a trial is consistent with the view that the DLS plays a more important role than the DMS in outcome evaluation in the present behavioral task. Note that our results (early/late activation of the DMS/DLS within a trial) are not related to the previous finding of early/ate activation of the DMS/DLS in the course of learning a new task^40^. The animals were overtrained before unit recording, and similar results obtained when we divided the behavioral data into two halves (early and late sessions) and repeated the same analyses (data not shown). It is unclear what functions higher firing of DMS neurons during the early task phase is related to. Choice and action value signals were elevated in the DMS after behavioral manifestation of the animal’s target selection (after approach onset), suggesting that they are unlikely to be related to action selection^22^ or response vigor^43^. Additional studies are needed to understand whether and how these neural signals contribute to value-based decision making under the requirement for temporal credit assignment. It would be also important to explore interactions of the striatum with other brain structures, such as the hippocampus^25^, in dealing with the temporal credit assignment problem. For example, the striatum might be able to bridge a relatively short temporal gap between an action and its outcome, whereas the hippocampus might be necessary to handle the temporal credit assignment problem when the temporal gap between an action and its outcome is relative long.

## Methods

### Subjects

Three adult male Sprague Dawley rats (~10–12 weeks old, 300–350 g) were individually housed in a colony room and initially allowed free access to food and water for one week. They were then subjected to water deprivation with free access to food and extensive handling for 5–7 d. Their body weights were maintained at >80% ad libitum throughout the experiments. Experiments were conducted in the dark phase of a 12 h light/dark cycle. All animal care and experimental procedures were performed in accordance with protocols approved by the directives of the Animal Care and Use Committee of the Korea Advanced Institute of Science and Technology (Daejeon, Korea).

### Behavioral task

The animals performed a dynamic TAB task in a Y-shaped maze (Fig. 1a). They were allowed to choose freely between two targets for water reward. Each trial began when the animal arrived at the delay position (‘D’ in Fig. 1a) from the reward location (proximal end of the maze; ‘R’ in Fig. 1a). The connecting bridge (distal segment of the central stem) was lowered 2 s following the animal’s arrival at the delay position, allowing the animal to move forward. When the animal’s snout broke the photobeam at either target site (distal parts of the Y-maze; ‘T’ in Fig. 1a), an auditory tone (left target, 4.3 kHz; right target, 4.5 kHz) was delivered during the period of photobeam-breaking to signal that a choice was successfully completed. The outcome of a choice was revealed only after the animal came back to the reward location (R) and waited there for 1 s. Trial outcome was also signaled by an auditory tone (100 ms, 1 and 9 kHz for positive and negative outcomes, different across rats) so that onset of water delivery and the positive auditory tone coincided. A new trial began as soon as the animal arrived at the delay position without an inter-trial interval.

Each session consisted of four blocks, and each block consisted of 40–75 trials (54.2 ± 6.5, mean ± SD; first block, 40 plus a number randomly chosen between 0 and 20; the other blocks, 45 plus a number randomly chosen between 0 and 30). The sequence of block reward probabilities (four combinations, 0.72:0.12, 0.12:0.72, 0.63:0.21, and 0.21:0.63) was randomly determined with the constraint that the higher reward probability target always changed its location across adjacent blocks. A fixed amount of water (30 μl) was delivered stochastically in each trial according to the block reward probability of the chosen target.

### Behavioral stages

We focused our analysis on five behavioral stages. The delay stage is the first 2 s period of each trial, during which the animal was waiting for the connecting bridge to be lowered. The animal’s arrival at the delay position (‘D’ in Fig. 1a) was detected by a photobeam sensor that triggered the lowering of the bridge after a 2-s delay. The approach stage is between the first behavioral manifestation of the animal’s choice (its approximate position is indicated by ‘A’ in Fig. 1a) and activating a photobeam sensor at either target site (T). For each session, left- and right-choice-associated head positions were aligned temporally to the offset of the delay stage, and the time point when the X-coordinates of the left- and right-choice trials first became significantly different (*t*-test, *p* < 0.05) and remained that way for at least 100 ms was determined as the onset of the approach stage^14^. The selection stage started with the animal’s activating a target photobeam sensor (i.e., offset of the approach stage) and ended when the animal’s left- and right-choice trajectories converged. The stem of the maze was narrow (5 cm) and long (70 cm), which made the animal’s body position independent of the animal’s target choice around the midpoint of the central stem (‘C’ in Fig. 1a). The memory stage is between the time of trajectory convergence and the time that the trial outcome was revealed. Thus, the memory stage included a 1 s waiting period at the reward location (‘R’ in Fig. 1a). The onset of the memory stage was determined separately for each session. For this, we temporally aligned left- and right-choice-associated head positions to the time point 3 s prior to the reward stage onset. The time point when the difference in x coordinates of the left- and right-choice trials first became statistically insignificant (*t*-test, *p* > 0.05) and remained that way for at least 100 ms was determined as the onset of the memory stage. The reward stage is between the offset of the memory stage (which coincided with the onset of a positive/negative auditory cue and water delivery in positive outcome trials) and the beginning of the next trial (i.e., onset of the next delay stage). The duration of the reward stage was 14.92 ± 0.43 and 6.43 ± 0.25 s in rewarded and unrewarded trials, respectively. Thus, the animal’s behavior was different depending on the choice outcome especially during the later phase of the reward stage.

### Models of behavior

We used a hybrid model containing simple RL and win-stay-lose-switch terms^44^,^45^,^46^. This model was superior to the models containing only the RL or win-stay-lose-switch term (smaller values of Akaike’s and Bayesian information criteria). For the chosen action ‘*a*’, the action value (*q*_a_(*t* + 1)) was updated as follows:


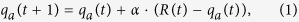


where *α* is the learning rate; and *R*(*t*) is the reward (1 if rewarded, and 0 otherwise) in the *t*-th trial. The total action value (*Q*_a_(*t* + 1)), including the action value and the win-stay-lose-switch term, was represented as follows:


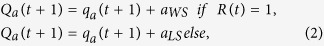


where *α*_*WS*_ and *α*_*LS*_ are win-stay and lose-switch terms, respectively. The action value for the unchosen action did not change across trials. Choices were made according to the softmax action selection rule as follows:


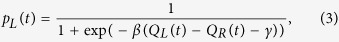


where *p*_L_(*t*) is the probability for selecting the left goal; *β* is the inverse temperature that determines the degree of randomness in action selection; and *γ* is a bias to select the right target. Model parameters for each rat are shown in Table 1.

### Unit recording

Single units were recorded simultaneously from the right DMS (center of the tetrode bundle, 0.5 mm anterior, and 2.0 mm lateral to bregma; 3.4–5.0 mm ventral to brain surface) and DLS (0.5 mm anterior and 4.0 mm lateral to bregma; 3.4–5.6 mm ventral to brain surface) with 15 tetrodes (7 or 8 in each region; Fig. 2a). For comparison with previous results, units were recorded in the same coordinates as in our previous study^8^. Tetrode fabrication and unit recording procedures are described in our previous study^14^. Briefly, unit signals were amplified 5000–10000×, band pass-filtered between 600–6000 Hz, digitized at 32 kHz, and stored on a personal computer using a Cheetah data acquisition system (Neuralynx). Unit signals were recorded with the animals placed on a pedestal for ~5 min before and after each experimental session to examine stability of recorded units. The head position of the rat was also recorded at 30 Hz by tracking an array of light emitting diodes mounted on the headstage. Small marking lesions were made by passing electrolytic current (30 μA, 20 s, cathodal) through one channel of each tetrode at the end of the final recording, and electrode tracks and lesions were verified under a fluorescent microscope as previously described^47^.

### Isolation and classification of units

Single units were isolated by manually clustering various spike waveform parameters using MClust software (A. R. Redish). The identity of a unit was determined based on the clustering pattern of spike waveform parameters, average spike waveforms, mean discharge rates, autocorrelograms, and inter-spike-interval histograms. Only those clusters with no inter-spike interval <2 ms, L-ratio <0.2, isolation distance >15^48^, and the number of spikes >500 were included in the analysis. Unstable units (magnitudes of average spike waveforms recorded before and after an experimental session differed by >10%) were also excluded from the analysis.

The recorded units were classified based on average firing rates and spike widths during the recording sessions according to our previous criteria^8^. Those units with mean firing rates <6 Hz and spike widths ≥0.24 ms were classified as putative MSNs, and the rest were classified as putative interneurons (Fig. 2b). Only putative MSNs (DMS: n = 212; DLS: n = 140) were included in the analysis. The mean ( ± SEM) firing rate and spike width of putative MSNs were 0.63 ± 0.05 Hz and 0.331 ± 0.003 ms in the DMS, respectively, and 0.81 ± 0.06 Hz and 0.331 ± 0.004 ms in the DLS, respectively.

### Multiple regression analysis

Neuronal activity related to the animal’s choice and its outcome was analyzed using the following regression model:


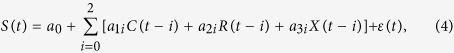


where *S*(*t*) indicates spike discharge rate in a given analysis window, *C*(*t*)*, R*(*t*), and *X*(*t*) represent the animal’s choice (left or right; dummy variable, −1 or 1), its outcome (reward or no reward; dummy variable, 1 or −1), and their interaction (choice × outcome; dummy variable, −1 or 1), respectively, in trial *t*, *a*_*0*_~*a*_*3*_ are regression coefficients, and *ε*(*t*) is the error term.

Neuronal activity related to action value and chosen value was analyzed with the following regression model:


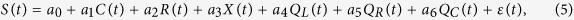


where *Q*_*L*_(*t*) and *Q*_*R*_(*t*) denote left and right total action values, respectively, and *Q*_*c*_(*t*) indicates the chosen value in trial *t* that was estimated using the hybrid model. Correlations among the regressors are summarized in Table 2.

### Statistical tests

Student’s *t*-tests (two-tailed) were used to examine the significance of a regression coefficient and the difference between left- and right-choice-associated head positions. A binomial test was used to test the significance of the fraction of neurons for a given variable, and a *χ*^2^-test was used to examine the significance of the difference in the fraction of neurons between the DLS and the DMS. A *p* value < 0.05 was used as the criterion for a significant statistical difference, and all data are expressed as mean ± SEM unless noted otherwise.

## Additional Information

**How to cite this article**: Her, E. S. *et al*. Neuronal activity in dorsomedial and dorsolateral striatum under the requirement for temporal credit assignment. *Sci. Rep*. **6**, 27056; doi: 10.1038/srep27056 (2016).

## Figures and Tables

**Table 1:** Parameters of the RL model for each rat. α, learning rate; *α*_*WS*_, win-stay coefficient; *α*_*LS*_, lose-switch coefficient; β, inverse temperature; *γ*, choice bias toward the right target.

	*α*	*α*_*WS*_	*α*_*LS*_	*β*	*γ*
Rat #1	0.033	−0.261	−0.334	1.794	0.180
Rat #2	0.072	−2.249	−2.555	0.349	0.505
Rat #3	0.106	0.318	−0.043	1.467	0.176

**Table 2:** Correlations among regressors.

	*C*(*t*)	*R*(*t*)	*X*(*t*)	*C*(*t* − 1)	*R*(*t* − 1)	*X*(*t* − 1)
*C*(*t*)	1	−0.008	−0.109	−0.091	−0.096	0.136
*R*(*t*)	−0.022	1	0.104	−0.041	7.43 × 10^−5^	0.021
*X*(*t*)	−0.120	0.106	1	0.065	0.051	0.268
*C*(*t* − 1)	−0.150	−0.033	0.063	1	−0.008	−0.109
*R*(*t* − 1)	−0.105	−0.023	0.059	−0.022	1	0.105
*X*(*t* − 1)	0.140	0.030	0.258	−0.118	0.107	1
	***C*(*t*)**	***R*(*t*)**	***X*(*t*)**	***Q*_*L*_(*t*)**	***Q*_*R*_(*t*)**	***Q*_*C*_(*t*)**
*C*(*t*)	1	−0.008	−0.109	−0.224	0.218	−0.071
*R*(*t*)	−0.022	1	0.104	0.001	0.052	0.107
*X*(*t*)	−0.120	0.106	1	−0.037	0.038	0.043
*Q*_*L*_(*t*)	−0.253	0.002	−0.020	1	−0.191	0.192
*Q*_*R*_(*t*)	0.253	0.049	0.022	−0.259	1	0.463
*Q*_*C*_(*t*)	−0.087	0.095	0.049	0.155	0.430	1

Shown are Pearson’s correlation coefficients between explanatory variables used in the multiple linear regression models. Top, regression model 1 (eq. 4); bottom, regression model 2 (eq. 5). Lower diagonal, DMS; upper diagonal, DLS.
